# Clinical efficacy and complications of blocking screw in the treatment of lower limb long bone fracture: An updated systematic review and meta-analysis

**DOI:** 10.1097/MD.0000000000037647

**Published:** 2024-04-05

**Authors:** Xiao Chen, Jing Chen, Chang Chen

**Affiliations:** a Department of Orthopedic Surgery, The First people’s Hospital of Neijiang, Neijiang, China; b Department of Neonatology, The First people’s Hospital of Neijiang, Neijiang, China.

**Keywords:** blocking screw, fracture, long bone, meta-analysis

## Abstract

**Background:**

Blocking screw technique has been widely applied in the treatment of long shaft fractures. However, the evidence with regard to whether intramedullary nail combined with blocking screw technique has better clinical efficacy over other is not clear. The aim of the study was to explore the clinical efficacy and complications of intramedullary nail combined with blocking screw technique in the treatment of femoral or tibial shaft fractures.

**Methods:**

The PuMed, Embase, OVID, Cochrane library, Web of Science, Wanfang, CNKI and Weipu data were searched for studies of intramedullary nail combined with blocking screw in treatment of femoral or tibial shaft fracture published up to Aug 31 2023. Methodological quality of the trials was assessed, relevant data were extracted, and RevMan 5.3 and Stata 15.0 software were used to perform the meta-analysis of parameters related to the consequences.

**Result:**

Twenty articles were included, including 1267 patients. Meta-analysis results showed that compared with the non-blocking screw group, the blocking screw group had longer operation time (WMD = 13.24; 95% CI = 5.68–20.79, *P* = .0006) and more intraoperative fluoroscopy times (WMD = 57.62; 95% CI = 25.82–89.42, *P* = .0002). However, the postoperative therapeutic response rate was higher (OR = 5.60; 95% CI = 2.10–14.93, *P* = .0006), postoperative ankle joint function was better (OR = 3.48; 95% CI = 1.20–10.13, *P* = .02), and fracture healing rate was higher (OR = 3.56; 95% CI = 1.43–8.89, *P* = .006), fracture healing time was shorter (WMD = −3.59; 95% CI = −4.96 to −2.22, *P* < .00001), intraoperative blood loss was less (WMD = −54.80; 95% CI = −88.77 to −20.83, *P* = .002), hospitalization time was shorter (WMD = −1.66; 95% CI = −2.08 to −1.24, *P* < .00001), and complications were less (OR = 0.38; 95% CI = 0.16–0.89, *P* = .01). There was no statistical significance in the range of motion of knee joint between the 2 groups (WMD = 10.04; 95% CI = −1.51 to 21.59, *P* = .09).

**Conclusions:**

Current evidence shows that intramedullary nail combined with blocking screw technique in the treatment of lower limb long bone fracture has the advantages of good clinical efficacy, high fracture healing rate, short fracture healing time, good joint function, less complications and so on, which is worthy of clinical recommendation.

## 1. Introduction

Lower limb long bone fracture is usually caused by violence, which is a common fracture type in orthopedics, and with the development of transportation industry and construction industry, its incidence is increasing year by year.^[1,2]^ Among them, the annual incidence of femoral shaft fracture is about 10/100,000,^[3]^ accounting for about 6% of total body fractures,^[4,5]^ and the annual incidence of tibial shaft fracture is about 15.7/100,000,^[6]^ accounting for about 10% of total body fractures.^[7]^

Femoral and tibial shaft fractures tend to displaced, and surgical treatment such as intramedullary nail or plate screw internal fixation is often needed to promote fracture healing and restore limb function. As we all know, intramedullary nail internal fixation has the advantages of minimally invasive, strong fixation, early underground functional exercise and high fracture healing rate. It has been widely used in clinical practice and gradually become the preferred method for surgical treatment of femoral and tibial shaft fractures.^[8–10]^ However, the intramedullary nailing technique also has its shortcomings. Due to the enlargement of the femoral and tibia distal medullary cavity, this method has the problems of distal fracture block angulation and lateral displacement, which leads to difficulty in reduction and poor force line of lower limbs, resulting in delayed fracture healing and other complications.^[11–15]^

Therefore, Kerttek et al^[16]^ proposed the combined intramedullary nail blocking technique in 1999, which aims to reduce the width of the shaft medullary cavity, thus limiting the swing of the main nail in the medullary cavity, providing effective three-point fixation, facilitating the auxiliary fracture reduction, and achieving the purpose of correcting the lateral and angular fracture displacement.^[17]^ It has effectively expanded the indication of internal fixation technique of intramedullary nail for treating shaft fracture.^[18]^

In recent years, more and more studies have applied intramedullary nailing combined with blocking nailing technique in the treatment of long shaft fractures, confirming the conclusions of Kerttek et al.^[12,19–22]^ A previous Chinese meta-analysis by our team reached similar conclusions. However, there are no relevant evidence-based medical studies abroad reported at present. Moreover, new clinical studies reporting outcomes on a larger number of patients. To provide support for clinical decisions, we conducted a systematic review and meta-analysis of currently available clinical studies evaluating the clinical efficacy and safety of intramedullary nailing combined with blocking nailing technique for the treatment of femoral and tibial shaft fractures.

## 2. Materials and methods

We followed the methods of our previously published meta-analyses in 2018^[23]^ and 2020.^[24]^

### 2.1. Data sources and search strategy

PuMed, Embase, OVID, Cochrane library, Web of Science, Wanfang, CNKI and Weipu data were searched for relevant randomized controlled trials, cohort studies or case-control studies until the end of Aug 31, 2023 related to the technique of blocking nailing of femoral and tibial shaft fractures and meeting the previously made inclusion and exclusion criteria. The following keywords were used to search the databases: “femur,” “femoral,” “tibial,” “tibia,” “diaphysis,” “shaft,” “fracture,” “blocking screw,” “poller screw,” “blocking nailing,” “trial.” Google Scholar was also searched to identify potentially relevant literature. In addition, reference lists of identified reports were reviewed for other potentially relevant studies.

### 2.2. Inclusion and exclusion criteria

Studies were selected based on the following inclusion criteria: randomized controlled trials (RCTs), cohort studies, or retrospective or prospective case-control studies of blocking screw (BS) for treatment of femoral or tibial shaft fracture without restrictions on time of publication or country of origin; patients with definite clinical diagnosis of femoral or tibial shaft fracture (age is not limited); The study group used intramedullary nailing combined with blocking screw technique, while the control group used non-blocking screw (NBS) technique, including intramedullary nailing alone, small plate attached to intramedullary nailing, open reduction plate internal fixation, MIPPO and other methods; outcome: operation time, intraoperative blood loss, intraoperative fluoroscopy time, hospitalization time, postoperative treatment rate, range of motion of knee joint, postoperative ankle joint function, fracture healing time, fracture healing rate, imaging result, complications; and publication in English or Chinese.

Exclusion criteria were study objective or intervention measures failed to meet the inclusion criteria; repeatedly published literature, animal study, human autopsy and other non-clinical trials, reviews, case report, conference papers and studies without use-able data; and duplicate data from another study.

### 2.3. Data extraction and quality evaluation

Two authors searched, screened and extraction the data from all eligible article independently, and any disagreements were resolved by discussion and consensus among the authors. Studies were then selected by reading the title, abstract and full text. If necessary, attempts were made to contact the authors for required data.

Extracted data included basic publication and cohort information, intervention measures, outcome indicators and results, and relevant factors for risk of assessment. We also evaluated the potential bias in all included studies. For non-randomized trials, the Newcastle–Ottawa Scale was used for bias assessment. For randomized controlled trials, evaluation criteria and methods followed the Cochrane Collaboration proposal. Appraisal criteria included random sequence generation, allocation concealment, blinding of participants and personnel, blinding of outcome assessment, incomplete outcome data, selective reporting, and other sources of bias. Each of these factors was recorded as low risk, unclear risk, or high risk.

The following information was extracted from all qualifying articles: general information (name of first author, publication year, region where the population resided, study type, sample size, mean ages, and interventions) and outcomes (as defined above).

### 2.4. Statistical analysis

The extracted data were pooled using Review Manager 5.3. The mean difference (MD) and odds ratio (OR) were introduced to evaluate the differences among interventions on continuous variables and dichotomous variables, respectively, and 95% confidence interval (CI) was calculated as well to examine the significance. Heterogeneity of the included studies was evaluated using Higgins *I*^2^. A random-effect model was used when apparent heterogeneity was detected (*I*^2^ ≥ 50% or *P* < .05). Otherwise, a fixed effect model was used (*I*^2^ < 50% or *P* ≥ .05).

### 2.5. Publication bias

Potential publication bias was judged by Egger’s tests. Sensitivity analysis was performed to evaluate the robustness of the combined data. A *P* value < .05 was regarded as statistically significant for all tests.

## 3. Results

### 3.1. Search results

**Figure 1.**
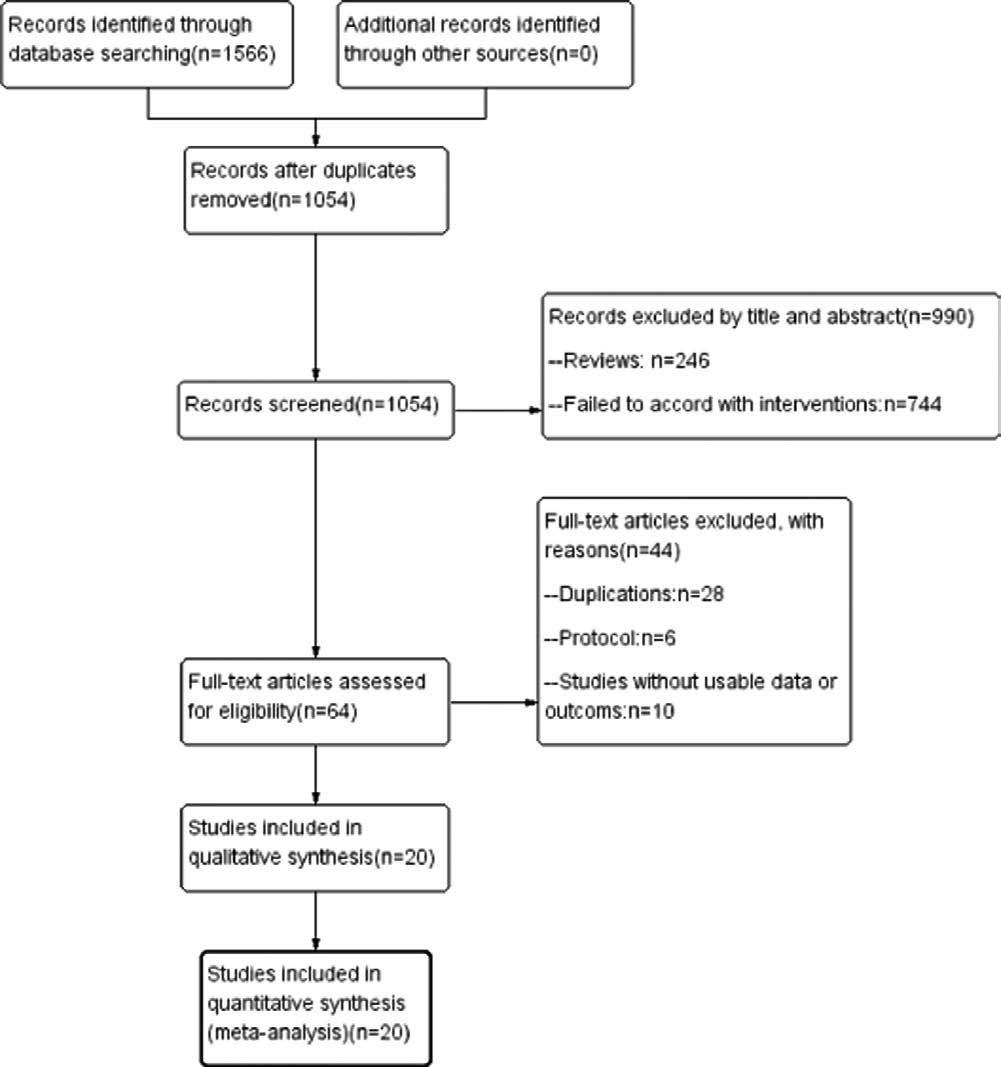
Flow chart for the process of screening out the included studies.

In accordance with the search strategy, 1566 publications were identified from electronic medical databases. After removing 512 duplicated studies, the remaining 1054 literature results were screened. Among them, only 64 met the inclusion standards. The remaining 990 other articles were reviews, animal-based studies, non-clinical trials, case report and conference papers. Furthermore, another 44 studies were excluded because of insufficient data or without use-able data. We included RCTs and non-RCTs because we required sufficient data to accomplish a good meta-analysis study. When only RCT articles were included, the number of references did not support the analysis. After reading the full text, 20 articles^[12,25–43]^ were finally included and used to provide primary data for further analysis, there were 4 randomized controlled trials,^[31–33,36]^ 16 case-control studies,^[12,25–30,34,35,37–43]^ 5 English literatures^[12,25–27,41]^ and 15 Chinese literatures,^[28–40,42,43]^ including 1267 patients. The study selection process and reasons for exclusion are summarized in Figure 1. And the main characteristics of these studies and patients are summarized in Table 1.

**Table 1:** Summary of study and patient characteristics.

Study	Country	Type	Intervention	No.	Age (year)	Outcome
Bryan 2018^[27]^	America	CCT	BS vs NBS	46	36.6 ± 15.1	(4)(5)(6)
				70	39.9 ± 17.7	
Adam 2019^[12]^	America	CCT	BS vs NBS	30	43 ± 18	(5)(6)
				54	41 ± 19	
Ross 2019^[25]^	Britain	CCT	BS vs NBS	10	22–61	(5)
				20	17–93	
Guo WY 2020^[29]^	China	CCT	BS vs NBS	17	36.9 ± 12.8	(1)(2)(3)(4)(5)
				19	34.1 ± 15.3	
Wang GH 2019^[37]^	China	CCT	BS vs NBS	26	40.5 ± 2.1	(3)(6)
				26	40.0 ± 2.3	
Huang WZ 2017^[31]^	China	RCT	BS vs NBS	42	36.7 ± 2.9	(1)(2)(3)(4)(6)
				42	37.2 ± 3.3	
Chen HQ 2021^[28]^	China	CCT	BS vs NBS	24	46.96 ± 2.36	(1)(2)(3)(5)(6)
				26	47.35 ± 2.15	
Li J 2020^[33]^	China	RCT	BS vs NBS	17	36.90 ± 12.80	(1)(2)(3)(4)(5)
				19	34.10 ± 15.30	
Huang YQ 2012^[32]^	China	RCT	BS vs NBS	26	41.7 ± 6.9	(1)(3)(4)(5)(6)
				26	42.0 ± 7.1	
Hou JZ 2016^[30]^	China	CCT	BS vs NBS	23	45.2 ± 17.4	(1)(3)(4)(6)
				28	47.5 ± 16.1	
Pan YF 2013^[35]^	China	CCT	BS vs NBS	31	18–61	(1)(3)
				24	21–63	
Zhang YY 2018^[40]^	China	CCT	BS vs NBS	35	45 ± 6.9	(3)
				21		
Yang ZY 2012^[39]^	China	CCT	BS vs NBS	17	20–67	(1)(3)(4)(6)
				15		
Meng LH 2021^[34]^	China	CCT	BS vs NBS	25	38.73 ± 12.85	(1)(2)(3)(4)(5)
				23	36.85 ± 12.77	
Wang B 2019^[36]^	China	RCT	BS vs NBS	21	64.62 ± 3.14	(3)(4)(6)
				21	64.58 ± 3.41	
Yang GH 2015^[38]^	China	CCT	BS vs NBS	18	43.85 ± 11.91	(1)(2)(3)(4)
				17		
Peat 2021^[41]^	Britain	CCT	BS vs NBS	88	/	(4)
				66		
Guo 2021^[26]^	China	CCT	BS vs NBS	33	43.9 ± 16.9	(1)(4)(6)
				63	50.2 ± 19.3	
Huang JP 2022^[43]^	China	CCT	BS vs NBS	26	65.36 ± 5.34	(1)(3)(4)
				26	64.72 ± 6.47	
Wei XJ 2022^[42]^	China	CCT	BS vs NBS	53	37.39 ± 5.44	(1)(3)(4)(5)(6)
				53	38.68 ± 5.29	

Outcome: (1) Intraoperative situation: including operation time, intraoperative blood loss, intraoperative fluoroscopy times, etc; (2) Postoperative conditions: including postoperative drainage, swelling regression time, length of hospital stay, etc; (3) Clinical efficacy: including effective rate (or excellent rate), knee range of motion, VAS score, ankle lowa excellent rate, ankle range of motion, etc; (4) Fracture healing: including fracture healing rate, fracture healing time, etc; (5) Imaging findings: coronal angle, sagittal angle, lateral displacement, etc; and (6) Complication.

BS = blocking screw, CCT = case-control trial, NBS = non-blocking screw, RCT = randomized controlled trial.

### 3.2. Quality assessment and basic information

**Figure 2.**
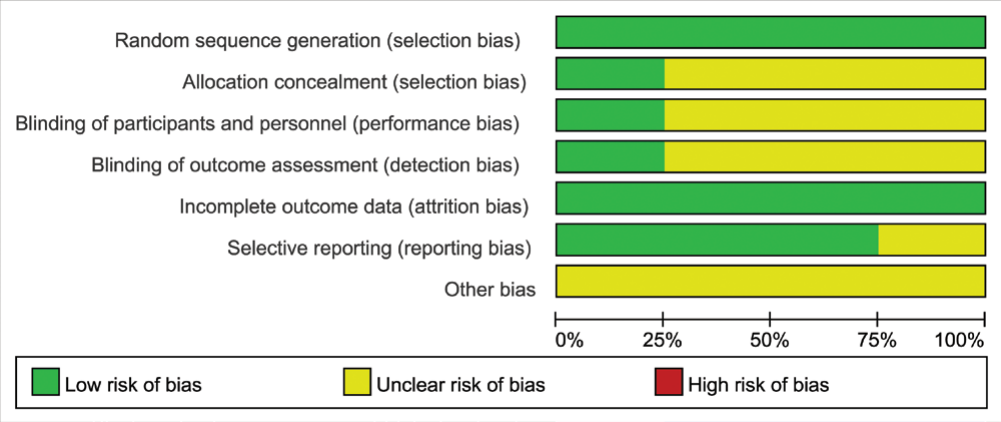
Risk of bias assessment summary.

**Figure 3.**
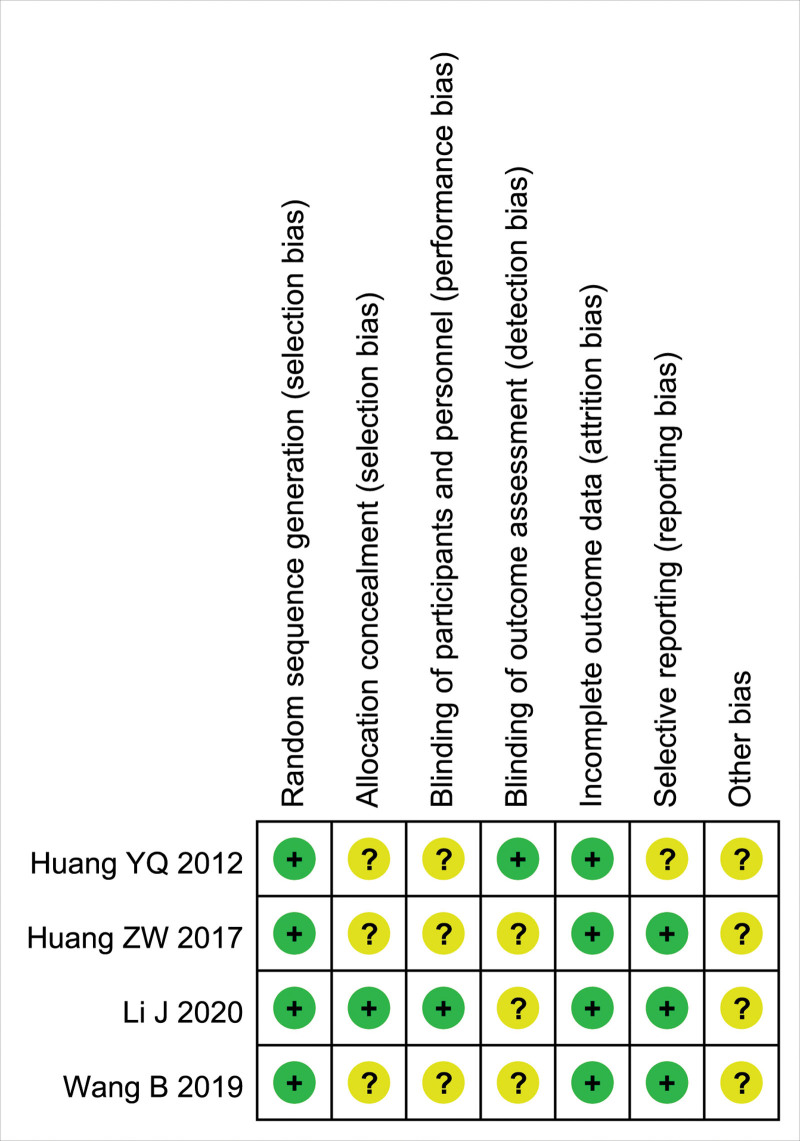
Risk of bias assessment summary.

The quality of the included RCTs was assessed using the Cochrane Collaboration’s “Risk of bias.” The risk of bias assessment of included studies is given in Figures 2 and 3. The risk of bias of the included non-RCTs evaluated with the Newcastle–Ottawa Scale score (the score ≥ 7 indicated good literature quality) is demonstrated in Table 2.

**Table 2:** The Newcastle–Ottawa scale score of non-RCT.

Study	1	2	3	4	5	6	7	8	Score
Bryan 2018^[27]^	1	1	0	1	2	1	1	1	8
Adam 2019^[12]^	1	1	0	1	2	1	1	1	8
Ross 2019^[25]^	1	1	0	1	2	1	1	1	8
Guo WY 2020^[29]^	1	1	0	1	1	1	1	1	7
Wang GH 2019^[37]^	1	1	0	1	1	1	1	1	7
Chen HQ 2021^[28]^	1	1	0	1	2	1	1	1	8
Hou JZ 2016^[30]^	1	1	0	1	2	1	1	1	8
Pan YF 2013^[35]^	1	1	0	1	1	1	1	1	7
Zhang YY 2018^[40]^	1	1	0	1	1	1	1	1	7
Yang ZY 2012^[39]^	1	1	0	1	2	1	1	1	8
Meng LH 2021^[34]^	1	1	0	1	1	1	1	1	7
Yang GH 2015^[38]^	1	1	0	1	1	1	1	1	7
Peat 2021^[41]^	1	1	0	1	2	1	1	1	8
Guo 2021^[26]^	1	1	0	1	2	1	1	1	8
Huang JP 2022^[43]^	1	1	0	1	1	1	1	1	7
Wei XJ 2022^[42]^	1	1	0	1	1	1	1	1	7

First item: Is the case definition adequate; Second item: Representativeness of the cases; Third item: Selections of Controls; Fourth item: Definition of Controls; fifth item: Comparability of case and controls on the basis of the design or analysis; sixth item: Ascertainment of exposure; seventh item: Same method of ascertainment for cases and controls; Eighth item: Non-response rate. Each item is 1 point except item 5, which has a value of 2 points.

RCT = randomized controlled trial.

### 3.3. Operation time

**Figure 4.**
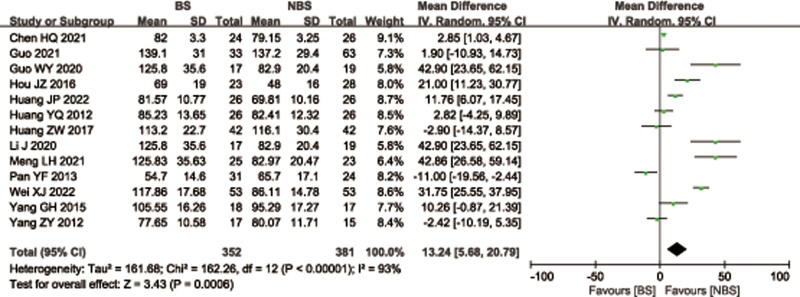
Forest plot of differences in operation time between BS and NBS groups. BS = blocking screw, NBS = non-blocking screw.

**Figure 5.**
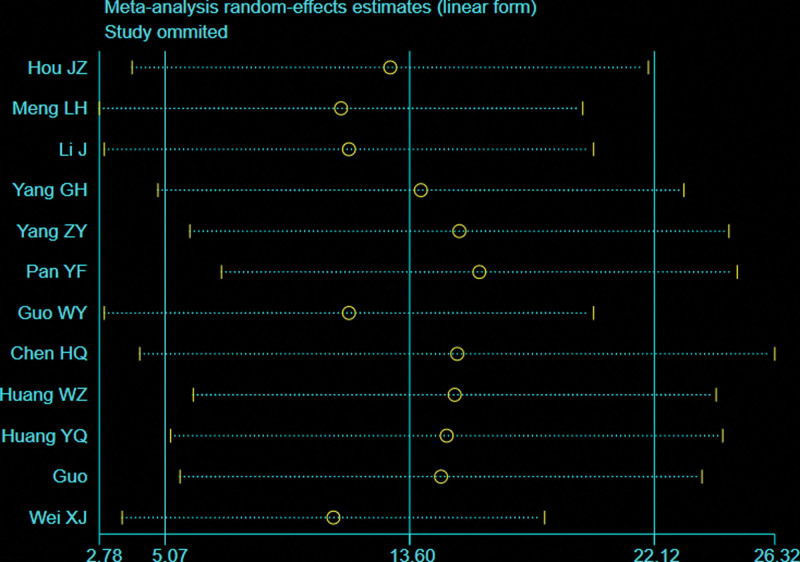
Sensitivity analysis of operation time.

Thirteen articles^[26,28–35,38,39,42,43]^ with 733 patients provide data on operation time for femoral or tibial shaft fractures. There was significant heterogeneity observed among studies (*I*^2^ = 93%, *P* < .00001), therefore, a random effects model was used. Data pooling revealed a significantly longer operation time in the BS group (weighted mean difference [WMD] = 13.24; 95% CI = 5.68–20.79, *P* = .0006; Fig. 4). Considering the significant heterogeneity, the Stata software was used for further sensitivity analysis, and the results showed that the total combined effect size of operation time didn’t change significantly after removing single study one by one, suggesting that the results were robust, as shown in Figure 5.

### 3.4. Intraoperative blood loss

**Figure 6.**
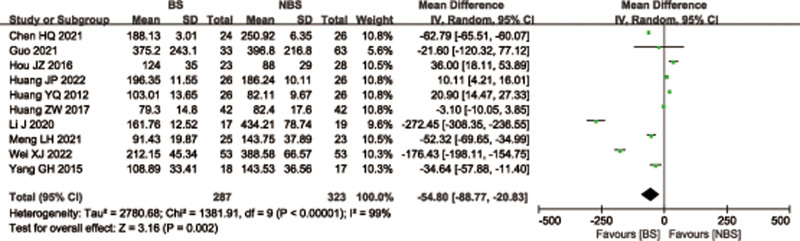
Forest plot of differences in intraoperative blood loss between BS and NBS groups. BS = blocking screw, NBS = non-blocking screw.

**Figure 7.**
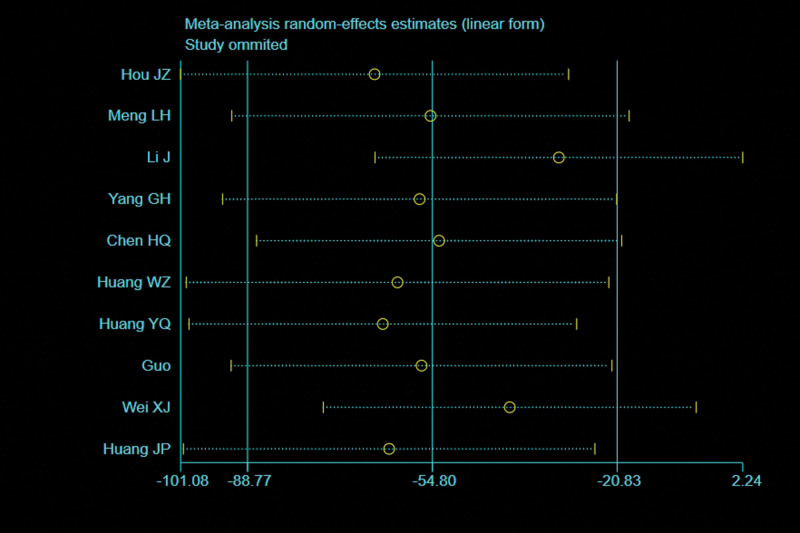
Sensitivity analysis of intraoperative blood loss.

A total of 10 studies^[26,28,30–34,38,42,43]^ with 610 patients reported intraoperative blood loss for femoral or tibial shaft fractures. There was significant heterogeneity observed among studies (*I*^2^ = 99%, *P* < .00001), therefore, a random effects model was used. The BS group demonstrated a significantly less intraoperative blood loss than the NBS group (WMD = −54.80; 95% CI = −88.77 to −20.83, *P* = .002; Fig. 6). Considering the significant heterogeneity, the Stata software was used for further sensitivity analysis, and the results showed that the total combined effect size of intraoperative blood loss didn’t change significantly after removing single study one by one, suggesting that the results were robust, as shown in Figure 7.

### 3.5. Intraoperative fluoroscopy times

**Figure 8.**
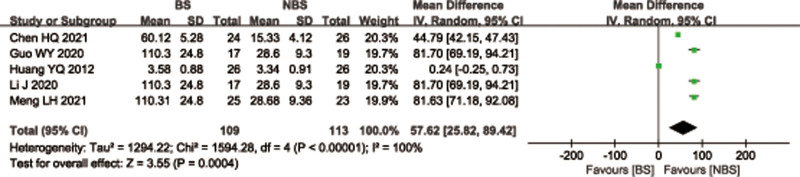
Forest plot of differences in intraoperative fluoroscopy times between BS and NBS groups. BS = blocking screw, NBS = non-blocking screw.

**Figure 9.**
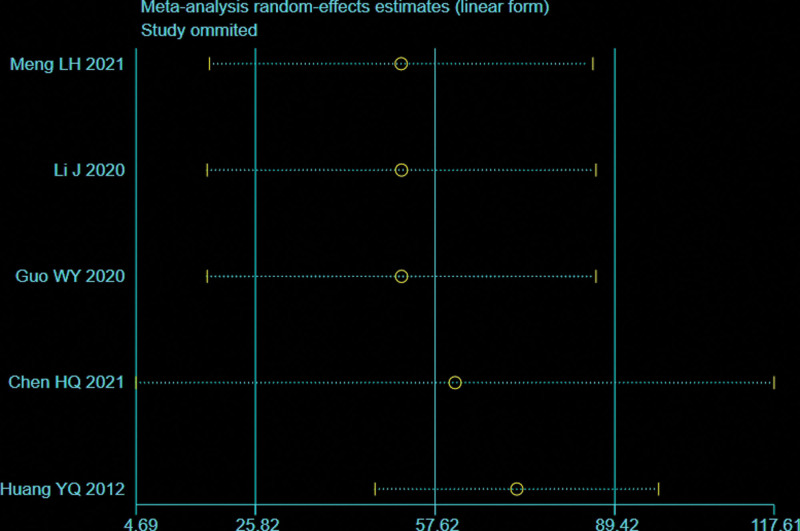
Sensitivity analysis of intraoperative fluoroscopy times.

Five trials^[28,29,32–34]^ reported the intraoperative fluoroscopy times (109 patients in the BS group and 113 in the NBS group). There was significant heterogeneity observed among studies (*I*^2^ = 100%, *P* < .00001), therefore, a random effects model was used. Data pooling indicated a significantly more intraoperative fluoroscopy times in the BS group (WMD = 57.62; 95% CI = 25.82–89.42, *P* = .0002; Fig. 8). Considering the significant heterogeneity, the Stata software was used for further sensitivity analysis, and the results showed that the total combined effect size of intraoperative fluoroscopy times didn’t change significantly after removing single study one by one, suggesting that the results were robust, as shown in Figure 9.

### 3.6. Postoperative swelling resolution time and hospitalization time

Only 2 articles^[31,38]^ reported postoperative swelling resolution time. There was no statistically significant differences between groups (WMD = −1.65; 95% CI = −5.34 to 2.03, *P* = .38; Table 3) with significant heterogeneity (*I*^2^ = 95%, *P* < .00001).

**Table 3:** Results of this meta-analysis.

Outcome	Number	Heterogeneity			Meta-analysis	
		*P*	*I* ^2^	Effect model	WMD/OR 95% CI	*P*
Postoperative swelling resolution time	2	<.00001	95%	Random	−1.65 (−5.34 to 2.03)	.38
Hospitalization time	4	.84	0%	Fixed	−1.66 (−2.08 to −1.24)	<.00001
Therapeutic response rate	4	.59	0%	Fixed	5.60 (2.10–14.93)	.0006
VAS	2	.17	48%	Fixed	0.17 (0.04–0.30)	.009
Lowa excellent and good rate	4	.22	33%	Fixed	3.48 (1.20–10.13)	.02
Range of motion of knee joint	3	.0003	88%	Random	10.04 (−1.51 to 21.59)	.09
Range of motion of ankle joint	2	.50	0%	Fixed	2.53 (0.81–4.25)	.004
Coronal angles	4	.78	0%	Fixed	0.57 (0.39–0.74)	<.00001
Sagittal angles	4	<.00001	98%	Random	1.08 (0.38–1.78)	.003
Lateral displacements	3	<.00001	97%	Random	0.49 (0.25–0.73)	<.0001

WMD = weighted mean difference.

Four studies^[29,33,34,42]^ provide data on hospitalization time for femoral or tibial shaft fractures. There was no significant heterogeneity observed among articles (*I*^2^ = 0%, *P* = .84), therefore, a fixed effects model was used. Data pooling revealed a significantly shorter hospitalization time in the BS group (WMD = −1.66; 95% CI = −2.08 to −1.24, *P* < .00001; Table 3).

### 3.7. Clinical efficacy

#### 3.7.1. Therapeutic response rate.

Four articles^[30,34,36,37]^ with 193 patients reported postoperative therapeutic response rate for femoral or tibial shaft fractures. There was no significant heterogeneity observed among articles (*I*^2^ = 0%, *P* = .59), therefore, a fixed effects model was used. Data pooling revealed a significantly higher postoperative therapeutic response rate in the BS group than NBS group (OR = 5.60; 95% CI = 2.10–14.93, *P* = .0006; Table 3).

#### 3.7.2. Visual analogue scale.

Only 2 trials^[28,34]^ provided data on VAS for femoral or tibial shaft fractures 6 months after surgery. A fixed effects model was used with low heterogeneity (*I*^2^ = 48%, *P* = .17). The pooled data showed significantly lower VAS in the BS group (WMD = 0.17; 95% CI = 0.04–0.30, *P* = .009; Table 3).

#### 3.7.3. Lowa excellent and good rate of ankle joint.

Four studies^[35,38,40,42]^ with 252 patients provided data on postoperative Lowa excellent and good rate of ankle joint for femoral or tibial shaft fractures. There was significant low heterogeneity observed among studies (*I*^2^ = 33%, *P* = .22), therefore, a fixed effects model was used. Data pooling indicated a significantly higher postoperative Lowa excellent and good rate of ankle joint in the BS group (OR = 3.48; 95% CI = 1.20–10.13, *P* = .02; Table 3).

#### 3.7.4. Range of motion of knee joint and ankle joint.

There were 3 trials^[28,29,33]^ reported the range of motion of knee joint for femoral or tibial shaft fractures. There was no statistically significant differences between groups (WMD = 10.04; 95% CI = −1.51 to 21.59, *P* = .09; Table 3) with significant heterogeneity (*I*^2^ = 88%, *P* = .0003). And only 2 articles^[32,39]^ provided data on the range of ankle join for femoral or tibial shaft fractures. There was no significant heterogeneity observed among articles (*I*^2^ = 0%, *P* = .50), therefore, a fixed effects model was used. Data pooling revealed a significantly better range of ankle join in the BS group than NBS group (WMD = 2.53; 95% CI = 0.81–4.25, *P* = .004; Table 3).

### 3.8. Fracture healing rate

**Figure 10.**
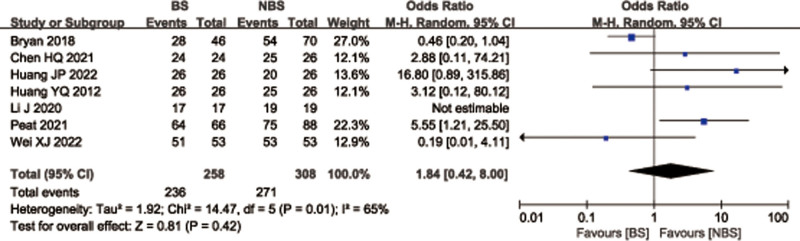
Forest plot of differences in fracture healing rate between BS and NBS groups. BS = blocking screw, NBS = non-blocking screw.

**Figure 11.**
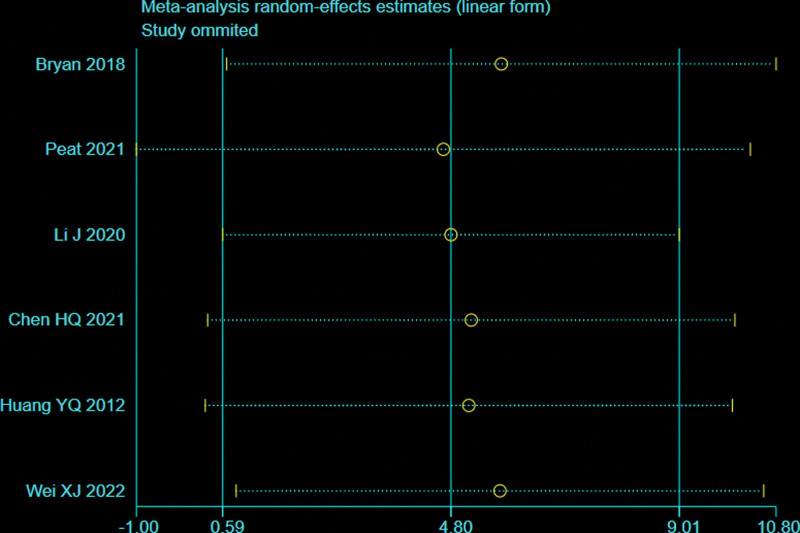
Sensitivity analysis of fracture healing rate.

**Figure 12.**
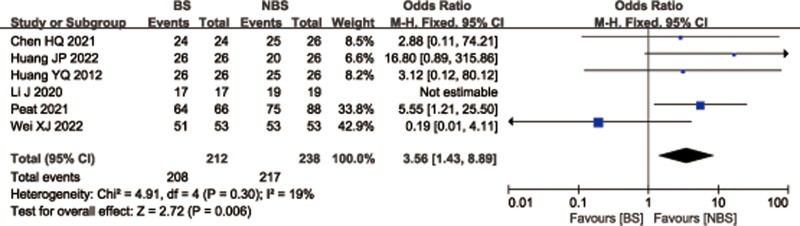
Forest plot of differences in fracture healing rate (after deletion of Bryan 2018 et al) between BS and NBS groups. BS = blocking screw, NBS = non-blocking screw.

A total of 7 studies^[27,28,32,33,41–43]^ with 566 patients reported fracture healing rate for femoral or tibial shaft fractures. There was no statistically significant differences between 2 groups (OR = 1.84; 95% CI = 0.42–8.00, *P* = .42; Fig. 10) with significant heterogeneity (*I*^2^ = 65%, *P* = .01). Considering the significant heterogeneity, the Stata software was used for further sensitivity analysis, and the results showed that heterogeneity and combined effect size of fracture healing rate changed significantly after deletion of Bryan 2018 et al^[27]^ study (Fig. 11). Meta-analysis was conducted on the remaining 6 studies.^[28,32,33,41–43]^ The heterogeneity of all studies was low (*P* = 19%, *P* = .30), therefore, a fixed effects model was used. And Data pooling indicated a significantly higher fracture healing rate in BS group than that in NBS group (OR = 3.56; 95% CI = 1.43–8.89, *P* = .006; Fig. 12).

### 3.9. Fracture healing time

**Figure 13.**
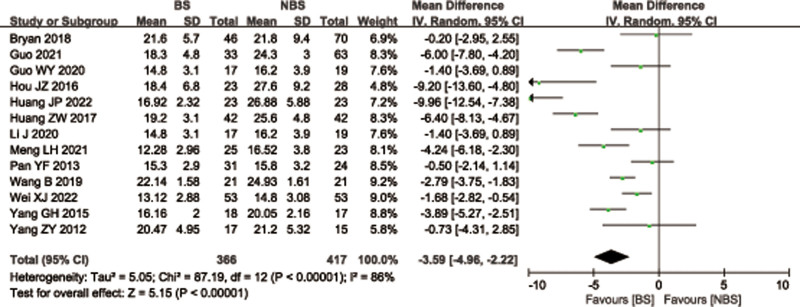
Forest plot of differences in fracture healing time between BS and NBS groups. BS = blocking screw, NBS = non-blocking screw.

**Figure 14.**
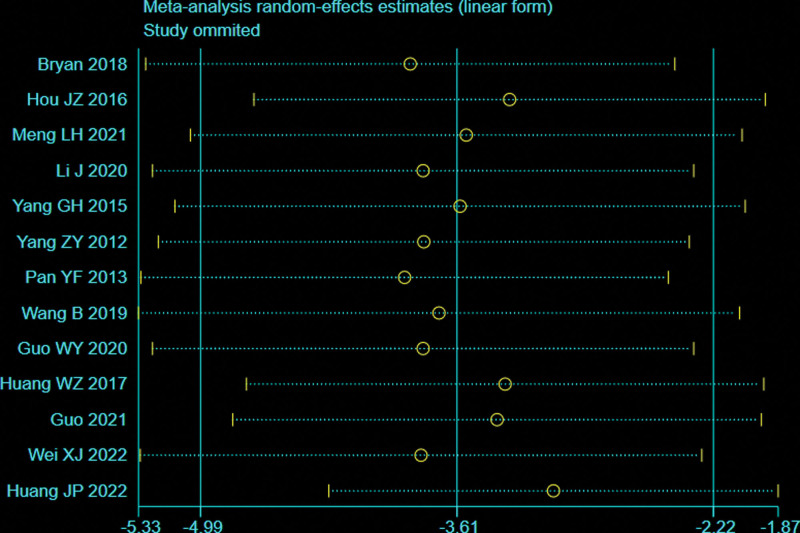
Sensitivity analysis of fracture healing time.

A total of 13 articles^[26,27,29–31,33–36,38,39,42,43]^ with 783 patients provided data on fracture healing time for femoral or tibial shaft fractures between 2 groups. There was significant heterogeneity observed among studies (*I*^2^ = 86%, *P* < .00001), therefore, a random effects model was used. Data pooling indicated a significantly shorter fracture healing time in the BS group than that of NBS group (WMD = −3.59; 95% CI = −4.96 to −2.22, *P* < .00001; Figure 13). Considering the significant heterogeneity, the Stata software was used for further sensitivity analysis, and the results showed that the total combined effect size of fracture healing time didn’t change significantly after removing single study one by one, suggesting that the results were robust, as shown in Figure 14.

### 3.10. Imaging results

Four trials^[29,33,34,42]^ reported coronal angles for femoral or tibial shaft fractures. There was no heterogeneity observed among studies (*I*^2^ = 0%, *P* = .78), and therefore, a fixed effects model was used. Data pooling indicated a significantly bigger coronal angles in the BS group (WMD = 0.57; 95% CI = 0.39–0.74, *P* < .00001; Table 3). And 4 articles^[29,33,34,42]^ provided data on sagittal angles for femoral or tibial shaft fractures. It showed that a significantly bigger sagittal angles in the BS group (WMD = 1.08; 95% CI = 0.38–1.78, *P* = .003; Table 3) with significant heterogeneity (*I*^2^ = 98%, *P* < .00001). Only 3 studies^[28,33,42]^ reported lateral displacements for femoral or tibial shaft fractures. And the result indicated that it was bigger significantly in the BS group (WMD = 0.49; 95% CI = 0.25–0.73, *P* < .0001; Table 3) with significant heterogeneity (*I*^2^ = 97%, *P* < .00001).

### 3.11. Complications

**Figure 15.**
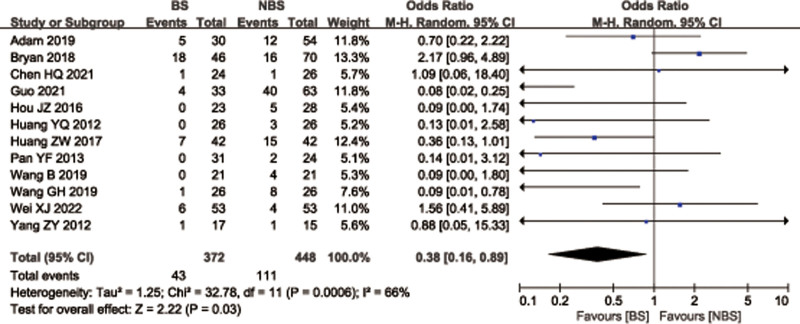
Forest plot of differences in complications between BS and NBS groups. BS = blocking screw, NBS = non-blocking screw.

**Figure 16.**
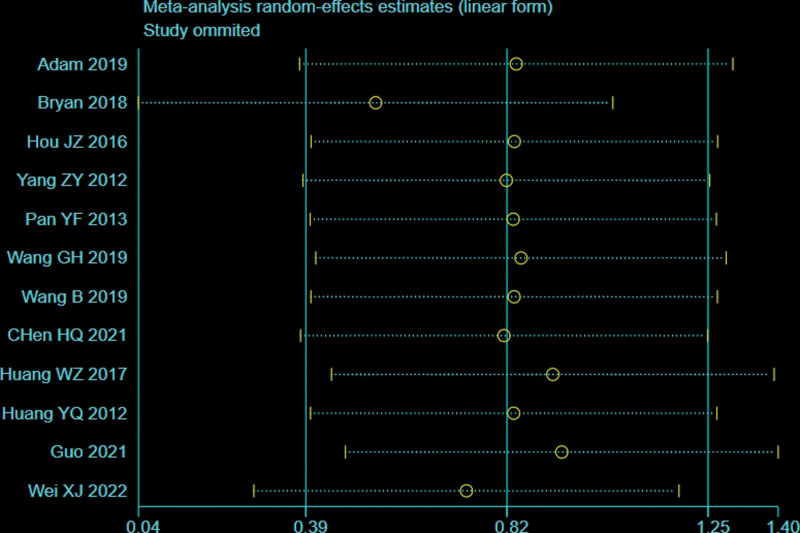
Sensitivity analysis of complications.

A total of 12 studies^[12,26–28,30–32,35–37,39,42]^ with 820 patients provided data on complications for femoral or tibial shaft fractures. There was significant heterogeneity observed among studies (*I*^2^ = 66%, *P* = .0006), therefore, a random effects model was used. Data pooling indicated a significantly low complications in the BS group than that of NBS group (OR = 0.38; 95% CI = 0.16–0.89, *P* = .01; Fig. 15). Considering the significant heterogeneity, the Stata software was used for further sensitivity analysis, and the results showed that the total combined effect size of complications didn’t change significantly after removing single study one by one, suggesting that the results were robust, as shown in Figure 16.

### 3.12. Publication bias

**Figure 17.**
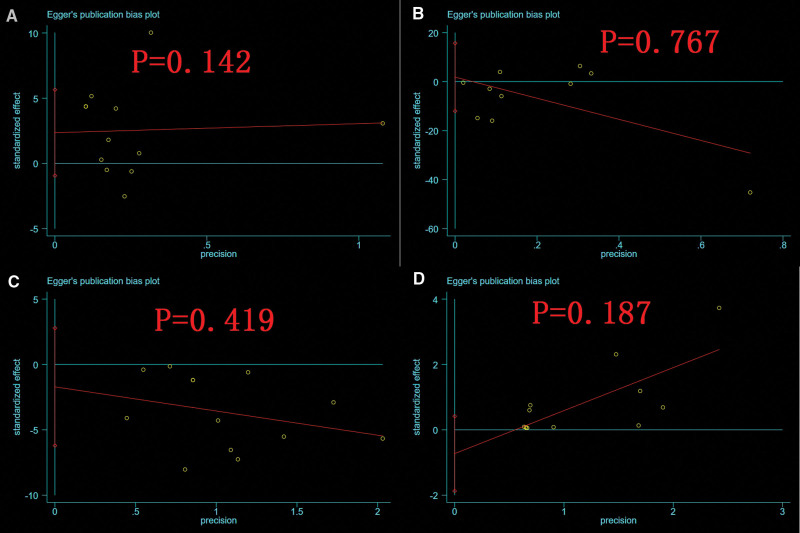
Egger’s publication bias plot of plot of operation time (A), intraoperative blood loss (B), fracture healing time (C), and complications (D) between BS and NBS groups. BS = blocking screw, NBS = non-blocking screw.

Egger test was used to analyze the publication bias of several indexes, such as operation time, intraoperative blood loss, fracture healing time and complications in the treatment of femoral and tibial shaft fracture by using intramedullary nailing combined with blocking screw technique. No significant publication bias was found and all *P* values were greater than .05 (Fig. 17).

## 4. Discussion

In recent years, intramedullary nailing has gradually become the standard surgical treatment for femoral and tibial shaft fractures.^[26]^ However, intramedullary nailing also has disadvantages, and studies have shown that it is associated with an increased risk of poor alignment or nonunion.^[44–46]^ Previous studies have shown that intramedullary nailing for 1/3 distal tibial fractures often leads to varus, torsion and nonunion,^[11,45,47]^ as does the femur.^[13,48,49]^ Zelle et al^[50]^reported in the early literature that the malunion rate of proximal tibial fractures treated with intramedullary nailing was as high as 84%, mainly for valgus and angulation. According to Freedman and Ricci et al,^[48]^ the incidence of misalignment in tibial fractures treated with intramedullary nailing was as high as 14% to 58%, and the incidence of femur fractures was as high as 10% to 30%. And other studies have shown that the rate of nonunion after treatment of tibial or femoral shaft fractures with intramedullary nailing is 5% to 25% and 0.5% to 12.5%, respectively.^[51–53]^ The use of blocking nail technology can effectively improve the fixation strength and force line of intramedullary nail fixation fracture, assist fracture reduction, correct lateral displacement and angular deformity. In order to verify the clinical efficacy of nail blocking technique in the treatment of femoral or tibial shaft fracture, scholars have carried out a large number of biomechanical tests and clinical trials, and obtained positive results, so as to guide the clinical application.

In this study, a total of 20 studies were included, and the meta-analysis results showed that despite longer operative time and more intraoperative fluoroscopy times with intramedullary nailing combined with nail blocking in the treatment of femoral or tibial shaft fractures, However, there was no statistical difference in knee range of motion with higher postoperative treatment efficiency (or excellent and good rate), better postoperative ankle joint function, higher fracture healing rate, shorter fracture healing time, less intraoperative blood loss, shorter hospital stay and fewer complications. There was significant heterogeneity in several indicators, such as operative time, intraoperative blood loss, intraoperative fluoroscopy times, fracture healing time and complications. Sensitivity analysis was conducted to exclude individual studies one by one, and no significant change was found in the total combined effect size, suggesting that the results of this study were robust. Egger test was used to analyze the publication bias of several indexes, such as operation time, intraoperative blood loss, intraoperative fluoroscopy times, fracture healing time and complications, in the treatment of femoral or tibial shaft fractures with and without block nails. The results showed that *P* values of all indexes were greater than .05, indicating that there was no significant publication bias in this study.

Chen et al^[28]^ conducted a comparative analysis of 50 patients with distal femoral fracture treated by intramedullary nail combined with blocking screw technique and open reduction and locking plate internal fixation technique, and found that the operative time and intraoperative fluoroscopy times of intramedullary nail-blocking technique were significantly longer than those of the plate group, with statistically significant differences. Meanwhile, A randomized controlled study carried out by Li et al^[33]^ compared the clinical efficacy of intramedullary nailing with small plate and blocking screw in the treatment of proximal tibial fractures, and also found that the blocking screw group had longer operation time and more intraoperative fluoroscopy times. The conclusion is similar to that of this study. The reason was considered to be that frequent X-ray fluoroscopy was required during the operation of blocking screw technology to find the most appropriate placement position of blocking screw, so the times of intraoperative fluoroscopy were increased and the operation time was prolonged. The average length of hospital stay, incision length, postoperative swelling and intraoperative blood loss of intramedullary nail combined with blocking screw were significantly lower than those of the non-blocking screw group. Analysis showed that the blocking screw group was minimally invasive surgery, requiring only a small incision for surgery, with less soft tissue damage. However, both the intramedullary nail plus small plate or the open reduction plate internal fixation group required incision for exposure and internal fixation, which damaged local skin veins, resulting in increased blood loss and affected blood return, longer surgical incision, and significant postoperative limb swelling, resulting in prolonged hospital stay.

The level of clinical efficacy is related to the degree of postoperative fracture reduction. In this study, the effective rate (or excellent and good rate), Lowa excellent and good rate, and ankle range of motion of the intramedullary nail combined with blocking screw in the treatment of tibial and femoral shaft fractures were significantly better than those of the non-blocking screw group. The fracture healing rate of the blocking screw group was significantly higher, and the fracture healing time was significantly shorter than that of the non-blocking screw group. Analysis the reason of the above phenomenon as follows: The distal femoral and tibial medullary cavity expands, and affected by the tendons, ligaments, or the surrounding soft tissue pull, when using intramedullary nail, possible deviation from intramedullary nail the nail axis backbone, leading to fracture end in sagittal and coronal in angulation deformity, is not conducive to fracture reset function, reducing the postoperative excellent and good rate. Before the application of blocking nail technology, in order to obtain a good lower limb force line and satisfactory reduction, physicians often choose to open reduction at the broken end of the fracture, and then perform intramedullary nail fixation after satisfaction, or directly perform open reduction plate screw internal fixation. These treatments all increased soft tissue injury to varying degrees, destroyed the blood supply of the fracture end, and also led to the loss of fibroblast growth factor (FGFs), bone morphogenetic protein (BMP), transforming growth factor-β (TGF-β) and other trace factors promoting fracture healing at the fracture end, increasing the risk of delayed fracture healing and nonunion. In addition, patients with open reduction plate internal fixation have late postoperative time to the ground, which is not conducive to early functional exercise, resulting in relatively poor knee or ankle joint function. A randomized controlled study by Wang et al^[36]^ found that the excellent and good rate of knee joint function in patients with distal femoral fracture treated by blocking screw technology combined with retrograde intramedullary nail was 95.42%, significantly higher than that in the open reduction plate group (71.43%), and the fracture healing time was significantly shorter than that in the plate group (*P* < .05). Meng et al^[34]^ analyzed the clinical efficacy of intramedullary nail combined with blocking screw in the treatment of 48 patients with proximal tibia fracture, and concluded that the excellent and good rate (92%) of blocking screw group was higher than that of non-blocking screw group(86.96%), and the VAS score was significantly lower than that of traditional group 1 and 3 months after surgery, but there was no significant difference in VAS score between the 2 groups half a year later. Meanwhile, the fracture healing rate of the blocking screw group was significantly higher, and the fracture healing time was significantly shorter than that of the non-blocking screw group, similar conclusions were drawn in our study.

Guo et al^[26]^ conducted a comparative analysis of 96 patients with femoral and tibial shaft fractures treated by intramedullary nailing combined with nail blocking technology, and found that the total complications in the 2 groups were 12.1% and 63.5% respectively. The total complications in the nail blocking group were significantly less than those in the control group (*P* = .08), which mainly focused on bone nonunion and secondary surgery. The incidence of postoperative nonunion in the 2 groups was 0% and 12.7% (*P* = .031), and the rate of reoperation was 3.0% and 19.0% (*P* = .031), respectively, which were significantly lower than that in the non-blocking nail group. Similar conclusions were drawn in this study. According to the research views of Yao et al,^[54]^ the principle of nail blocking technology is that the inserted nail is perpendicular to the coronal or sagittal plane of the intramedullary nail, which reduces the space of the shaft medullary cavity. Meanwhile, during reduction, the main nail of the intramedullary nail can be guided to be inserted into the distal center in the correct direction, and the swing of the main nail of the intramedullary nail can be prevented postoperatively, thus playing a mechanical role of three-point fixation. Thus achieve stable fracture end, correct angulation deformities, such as purpose, to obtain a good line of force and fracture.

The severity of femur or tibial shaft fracture, fracture type, age, and whether patients are complicated with other diseases may all influence the results, but due to the limited number of literatures, further subgroup analysis is not possible. Most of the subjects were tibial fracture patients, and there were too few studies on femoral fracture, which made it impossible to conduct subgroup analysis on femoral fracture and tibial fracture. The intervention measures of the control group included in the study were not completely the same, including traditional intramedullary nail, intramedullary nail combined with small plate, open reduction plate internal fixation and MIPPO, etc., which all affected the outcome indicators to a certain extent. Most of the included literatures are Chinese, and the research objects are mainly yellow people. There are few studies on other ethnic groups, and the evidence is slightly limited. Most of the literatures included in this study were non-randomized controlled studies, and the evidence strength was slightly weak. Meanwhile, the evaluation indexes of the literatures included were relatively incomplete.

## 5. Conclusion

Current evidence shows that intramedullary nail combined with blocking screw technique in the treatment of lower limb long bone fracture has the advantages of good clinical efficacy, high fracture healing rate, short fracture healing time, good joint function, less complications and so on, which is worthy of clinical recommendation. Of course, more large sample size and high-quality randomized controlled studies are needed for further validation, so as to provide more evidence-based medical evidence for clinical treatment of femoral and tibial fractures with intramedullary nailing combined with nail blocking technology.

## Author contributions

**Conceptualization:** Xiao Chen, Jing Chen.

**Data curation:** Xiao Chen, Jing Chen, Chang Chen.

**Formal analysis:** Xiao Chen.

**Funding acquisition:** Xiao Chen.

**Investigation:** Xiao Chen, Jing Chen, Chang Chen.

**Methodology:** Xiao Chen, Jing Chen, Chang Chen.

**Project administration:** Xiao Chen.

**Resources:** Xiao Chen.

**Software:** Xiao Chen, Jing Chen, Chang Chen.

**Supervision:** Xiao Chen, Jing Chen.

**Validation:** Xiao Chen.

**Visualization:** Xiao Chen.

**Writing – original draft:** Xiao Chen, Jing Chen.

**Writing – review & editing:** Xiao Chen, Jing Chen, Chang Chen.
